# Traumatic Pancreatic Injury Presentation, Management, and Outcome: An Observational Retrospective Study From a Level 1 Trauma Center

**DOI:** 10.3389/fsurg.2021.771121

**Published:** 2022-01-28

**Authors:** Hassan Al-Thani, Ahmed Faidh Ramzee, Ammar Al-Hassani, Gustav Strandvik, Ayman El-Menyar

**Affiliations:** ^1^Department of Surgery, Trauma Surgery Section, Hamad General Hospital (HGH), Doha, Qatar; ^2^Clinical Research, Trauma and Vascular Surgery, Hamad General Hospital, Doha, Qatar; ^3^Clinical Medicine, Weill Cornell Medical College, Doha, Qatar

**Keywords:** pancreas, trauma, blunt abdomen trauma, injury, visceral

## Abstract

**Background:**

We aimed to study the presentation, management, and outcomes of patients with a pancreatic traumatic injury.

**Methods:**

We retrospectively analyzed data for all patients who were admitted with pancreatic injuries between 2011 and 2017 at the only level 1 trauma center in the country.

**Results:**

There were 71 patients admitted with pancreatic trauma (0.6% of trauma admissions and 3.4% of abdominal injury admissions) with a mean age of 31 years. Sixty-two patients had pancreatic injury grade I–II and nine had injury grade III–IV. Thirty-eight percent had Glasgow Coma Scale (GCS) <9 and 73% had injury Severity Score (ISS) >16. The level of pancreatic enzymes was significantly proportional to the grade of injury. Over half of patients required laparotomy, of them 12 patients had an intervention on the pancreas. Eight patients developed complications related to pancreatic injuries ranging from pancreatitis to pancreatico-cutaneous fistula while 35% developed hemorrhagic shock. Mortality was 31% and regardless of the grade of injury, the mortality was associated with high ISS, low GCS, and presence of hemorrhagic shock.

**Conclusion:**

Pancreatic injuries following blunt trauma are rare, and the injured subjects are usually young men. However, most injuries are of low-grade severity. This study shows that regardless of the pancreatic injury grade on-admission shock, higher ISS and lower GCS are associated with worse in-hospital outcomes. Non-operative management (NOM) may suffice in patients with lower grade injuries, which may not be the case in patients with higher grade injuries unless carefully selected.

## Introduction

Pancreatic injury following abdominal trauma is a rare entity, however, it is usually associated with other visceral injuries and entails significant morbidity and mortality. The morbidity and mortality rates vary as 23.4–53% and 17.5–70%, respectively (1–3).

The integrity of the pancreatic duct is an important factor for the appropriate decision-making and prognosis after pancreatic injury (4). Pancreatic injuries often constitute a major diagnostic and therapeutic challenge. A high index of suspicion is necessary as the retroperitoneal location of the pancreas contributes to a delay in the presentation of signs, symptoms, and biochemical changes in the initial stages of injury (3, 5). The incidence of pancreatic injury has been reported in 0.4–3.6% of all trauma admissions and 3.7–11% in patients with abdominal trauma (1, 6–10). There is an epidemiological variation worldwide with relation to the mechanism of injury (blunt vs. penetrating) owing to social and cultural differences; with most regions reporting a higher number of blunt injury in contrary to regions where easy access to firearms results in a larger cohort of penetrating injuries as in South Africa and the United States (USA) (2).

Initial diagnosis of pancreatic injuries may not be straightforward as the clinical and radiological signs may be initially subtle, and this may contribute to a delay in the diagnosis and treatment. Contrast-enhanced CT scan is the fastest and most comprehensive technique for detecting suspected pancreatic injuries and is the modality of choice in hemodynamically stable patients. Contrast-enhanced CT scan has high specificity (90–95%) but low sensitivity (52–54%) for ductal involvement. Up to 40% of pancreatic injuries can be missed or misdiagnosed (11–13). Pancreatic injuries become more evident 12–24 h after trauma (14).

Many scoring systems are available for defining categories of pancreatic injuries, however, the most widely accepted grading system is the Organ Injury Scaling (OIS) developed by the American Association for the Surgery of Trauma (AAST). With this system, typically, higher grade injuries correlate with higher mortality and complications (8). Recently, the World Society of Emergency Surgery and the AAST expert panel released guidelines for the management of duodenal, pancreatic, and extrahepatic biliary tree trauma and provided a classification system, which combines the AAST-OIS classification with the hemodynamic status of patients (9).

There is a lack of information on pancreatic trauma in our region in the Arab Middle East. Thus, we aimed to review our institution's experience of pancreatic injuries in adult subjects in terms of presentation, management, and outcomes.

## Materials and Methods

A retrospective analysis was conducted for prospectively maintained data of all patients who were admitted with pancreatic injuries post-trauma and managed at the only level I trauma tertiary facility in the country between 2011 and 2017. Records of all patients with pancreatic injury were reviewed and data pertaining to the following were gathered; demographics, and mechanism of injury, Glasgow Coma Scale (GCS), hemodynamic parameters, amylase and lipase levels, associated injuries, Injury Severity Score (ISS), Abbreviated Injury Score (AIS), pancreatic injury grade (AAST-OIS), imaging interventions (ultrasonography, CT scan, and MRI), laboratory tests (serum amylase and lipase), which has been done within the first 24 h of admission, hospital length of stay, complications, and management (non-operative management (NOM) and surgical intervention). On arrival to the trauma room in the emergency department, all patients underwent thorough clinical assessment and resuscitation according to Advanced Trauma Life Support (ATLS) guidelines. Our routine trauma investigations include plain chest and pelvic x-ray followed by pan-CT scan and later on, we may consider follow-up CT scan and or magnetic resonance cholangiopancreatography (MRCP) depending on the clinical evaluation and complexity of the injury, finally, we may consider endoscopic retrograde cholangiopancreatography (ERCP) as a diagnostic and therapeutic tool, if indicated. We excluded pediatric patients and those who died at the scene, on arrival to the hospital, or before having evidence of pancreatic injury.

The NOM includes **(**1) initial nil per os (NPO) with or without nasogastric tube insertion. (2) According to the clinical assessment and biochemical markers, PO feeding will start with clear fluid and gradually, if tolerated, proceed with full diet. (3) In some cases with evidence of pancreatitis, fat-free diet will be started. (4) If there are no signs of improvement, further investigation with MRCP to rule out complications that may require intervention and to study the integrity of pancreatic duct anatomy. (5) In some cases with a pancreatic leak or severe pancreatitis, a short-term octreotide therapy will be added.

This study obtained ethical approval from the Research Ethics Committee, at Medical Research Center, Hamad Medical Corporation (HMC), Doha, Qatar (IRB#14409/14& IRB # MRC-01-18-003) with a waiver of consent as there was no direct contact with patients and de-identified data were collected retrospectively.

### Statistical Analysis

Data were presented as proportion, median and range, or mean ± SD, as appropriate. Differences in categorical variables (grades I, II, III vs. VI), (ISS <16 vs. ISS >16), (GCS <9 vs. GCS >9), and hemorrhagic shock vs. no hemorrhagic shock were analyzed using the Chi-Square test. The normality of continuous variables was checked by the Kolmogorov-Smirnov test. Continuous variables were compared using Student's *t*-test for two groups or one -way ANOVA test for >2 groups, for parametric data. Mann-Whitney U test and Kruskal-Wallis test were used for non-parametric data, whenever applicable. A two-tailed *p* of < 0.05 was statistically significant. All data analyses were carried out using the Statistical Package for the Social Sciences, version 18 (SPSS, Inc., Chicago, IL, USA).

## Results

The study cohort included 71 patients with pancreatic injury (0.6% of total trauma admissions and 3.4% of total abdominal injury admissions) of which 69 (97.2%) were males. Patients' age ranged from 18.6 to 43 years (mean 31 years). Most cases sustained polytrauma. The blunt injury was the most predominant type of trauma (91.5%); while only six patients had penetrating injuries. Among blunt trauma, motor vehicle collisions were the most common mechanism of injury followed by fall from height.

Table 1 shows patients' demographics, clinical presentation, associated injuries, procedures, complications, and outcomes in patients with pancreatic injury. The mean abdominal AIS was 2.9 ± 0.9, pancreatic injury scale was 1.72 ± 0.86, and ISS score was 27.2 ± 14.5.

**Table 1:** Demographics, clinical presentation, associated injuries, procedures, complications and outcomes in patients sustained pancreatic injury (*n* = 71).

**Variable**	**Value**	**Variable**	**Value**
**Age (mean** **±SD)**	30.8 ± 12.2	**Pancreatic head**	25 (35.2%)
**Males**	69 (97.2%)	**Pancreatic tail**	27 (38.0%)
**Trauma type**		**Pancreatic body**	22 (31.0%)
Blunt	65 (91.5%)	**Peripancreatic hematoma**	26 (36.6%)
Penetrating	6 (8.5%)	Intubated ETT	44 (62.0%)
**GCS at ED**; median (range)	14 (3–15)	CT scan abdomen	71 (100%)
**Serum lipase**	84 (13–2,057)	FAST (positive)	34 (47.9%)
**Serum amylase**	53 (8–1,107)	**Procedures**	
**Associated injuries**		Chest tube insertion	36 (50.7%)
Head	30 (42.3%)	Exploratory laparotomy	37 (52.1%)
Lung contusion	24 (33.8%)	Open reduction internal fixation	12 (16.9%)
Pneumothorax	20 (28.2%)	Spinal surgery	3 (4.2%)
Hemothorax	13 (18.3%)	Thoracotomy	4 (5.6%)
Hemo-pneumothorax	6 (8.5%)	**Pancreatic injury complications**	8 (11.3%)
Rib fracture	27 (38.0%)	Collection had CT-guided drainage	4 (50.0%)
Spleen	23 (32.4%)	Pancreatitis	2 (25.0%)
Liver	21 (29.6%)	Pseudocyst	1 (12.5%)
Small Bowel	8 (11.3%)	Pancreatic cutaneous fistula	1 (12.5%)
Mesentery	11 (15.5%)	**Hospital length of stay**	14 (1–61)
Kidney	11 (15.5%)	**ICU length of stay**	6 (1–39)
Stomach	5 (7.0%)	**Ventilatory days**	8 (1–30)
Mesenteric vein	3 (4.2%)	Blood transfusion	51 (71.8%)
Inferior vena cava	1 (1.4%)	Blood units transfused	8 (1–32)
Aortic injury	0 (0.0%)	**In-hospital complications**	
Diaphragmatic injury	3 (4.2%)	Wound Infection	11 (15.5%)
Retroperitoneal hematoma	18 (25.4%)	Pneumonia	11 (15.5%)
**Injury severity**		Acute respiratory distress syndrome	5 (7.0%)
Head AIS	4.3 ± 0.9	Sepsis	9 (12.7%)
Chest AIS	2.9 ± 0.7	Acute renal failure	3 (4.2%)
Abdomen AIS	2.9 ± 0.9	Coagulopathy	1 (1.4%)
Pelvis AIS	2.3 ± 0.6	**Mortality** ^*^	22 (31.0%)
Injury severity score	27.2 ± 14.5		
**Pancreatic laceration**	19 (26.8%)		
**Pancreatic contusion**	56 (78.9%)		
**Prominent pancreatic lobulation**	2 (2.8%)		
**Bulky pancreas**	1 (1.4%)		

* *20 head injury, 1 associated vascular injury, 1 multiorgan failure*.

Eight patients had isolated pancreatic injuries. Most patients had pancreatic contusions (*n* = 56) and the location of injury was almost evenly distributed between the head, body, and tail of the pancreas.

### Pancreatic Injury Grading

**Figure 1:**
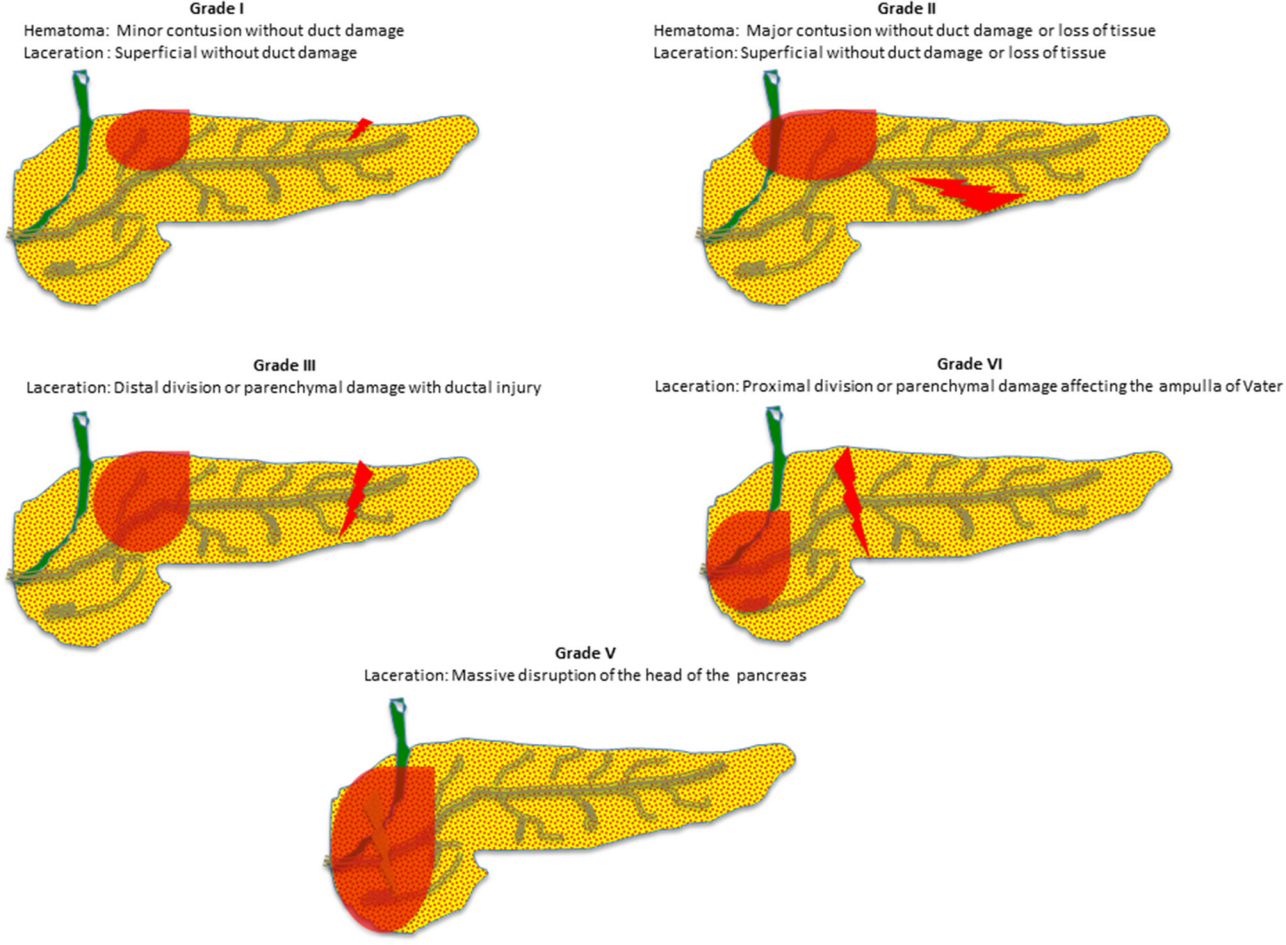
Illustration of pancreatic injury grades **(I–V)**.

Figure 1 shows an illustration of pancreatic injury grades (I–V). Peripancreatic hematomas were present in 36.6% of patients. Associated extra-abdominal injuries were mainly head and chest (42% and 38, respectively) that were mostly seen with low-grade pancreatic injuries.

Biochemical marker levels (amylase and lipase) revealed average levels of 53 and 84 U/l, respectively, but the degree of elevation was significantly proportional to the grade of pancreatic injury. Almost 17% of patients were found to be under the influence of alcohol, with average ethanol levels of 35.6 mmol/l.

Focused assessment with sonography in trauma (FAST) scan was positive in around half of the patients and CT scan of the abdomen was done in every case. Blood transfusion was required in 51 of the 71 patients with an average of 8 units transfused. Eight out of the 71 patients suffered from complications related to the pancreatic injuries (i.e., collections, pancreatitis, pseudocyst, and fistula). In-hospital complications are given in Table 1 (i.e., pneumonia, sepsis, organ failure, and coagulopathy).

**Figure 2:**
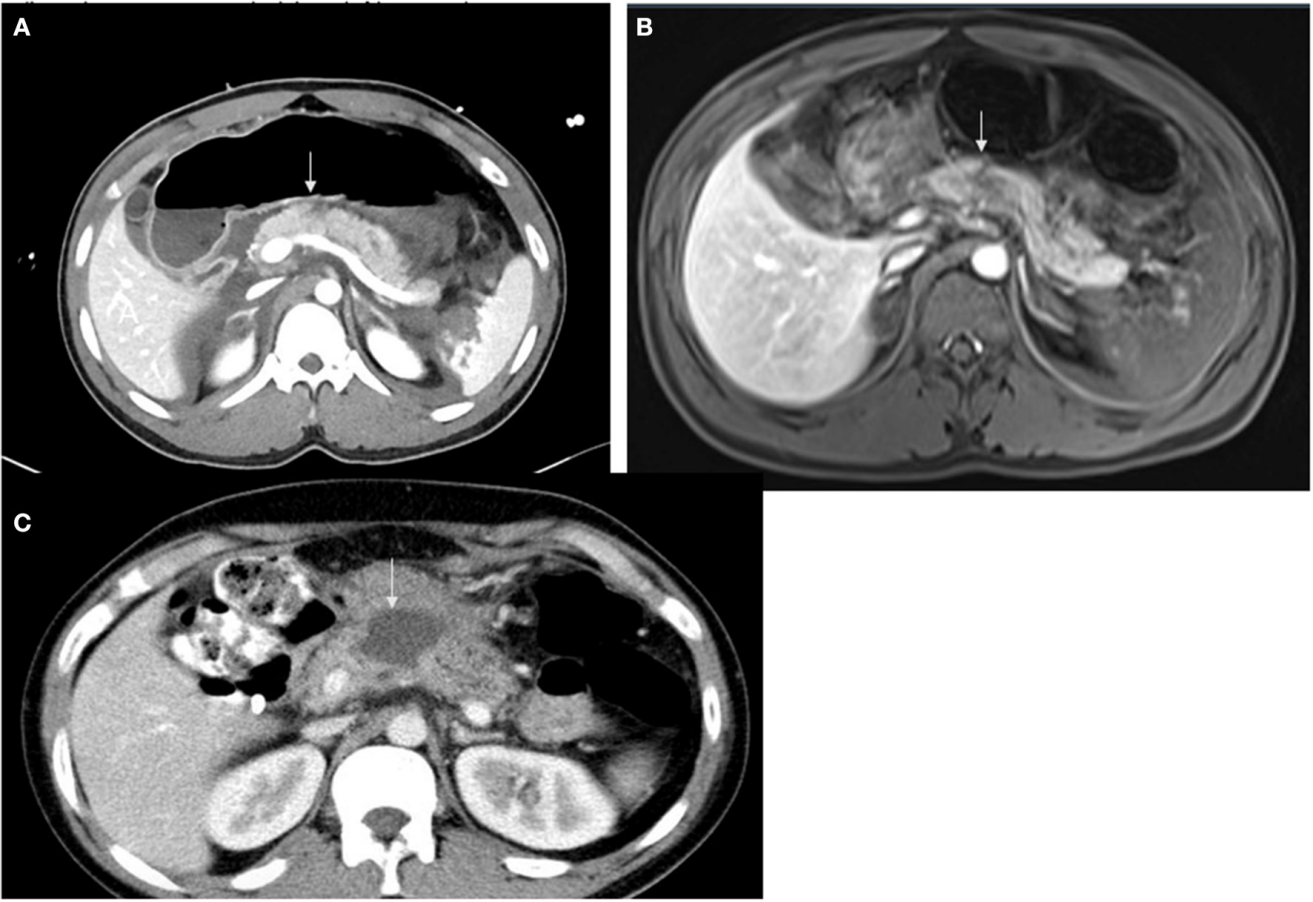
Examples of radiologic findings of pancreatic injury in the study cohort using CT scan and MRI. **(A)** Axial CT in a patient with pancreatic laceration noted at the superior aspect of the body anterior to the spine involving approximately 50% of pancreatic craniocaudal dimension (white arrows), **(B)** Axial MRI (same finding) T2: the junction between the head and body of the pancreas shows no enhancement in the dynamic phase representing pancreatic contusion. The pancreatic duct adjacent to the contusion area displays focal dilatation without ductal transection. **(C)** Axial CT shows pseudocyst.

Table 2 shows demographics, clinical presentation, and associated injuries by pancreatic injury scale according to the AAST. Low-grade pancreatic injuries were predominant, accounting for 87.3% of all patients (34 had grade I and 28 had grade II injuries) while four patients had grade III and five had grade IV injuries. There was no patient with grade V pancreatic injuries in this cohort. Figure 2 shows examples of radiologic findings of pancreatic injury in the study cohort using CT scan and MRI.

**Table 2:** Demographics, clinical presentation and associated injuries by pancreatic injury scale.

	**Grade I (*n* = 34)**	**Grade II (*n* = 28)**	**Grade III (*n* = 4)**	**Grade IV (*n* = 5)**	***P*-value**
**Age (mean** **±SD)**	29.5 ± 12.4	34.5 ± 11.7	20.0 ± 10.9	28.8 ± 8.2	0.10
**Males**	33 (97.1%)	27 (96.4%)	4 (100%)	5 (100%)	0.95
**GCS ED**	14 (3–15)	14 (3–15)	15 (15–15)	15 (3–15)	0.19
**Serum lipase**	59 (13–737)	137 (14–624)	747 (268–1,191)	674 (84–2,057)	0.001
**Serum amylase**	43 (8–394)	58.5 (15–254)	237 (72–462)	249 (40–1,107)	0.006
**Associated injuries**					
Head	16 (47.1%)	13 (46.4%)	1 (25.0%)	0 (0.0%)	0.19
Thoracic	21 (61.8%)	13 (46.4%)	3 (75.0%)	3 (60.0%)	0.54
Intraabdominal	26 (76.5%)	21 (75.0%)	3 (75.0%)	4 (80.0%)	0.99
**Head AIS**	4.7 ± 0.9	4.0 ± 0.9	3.0 ± 0.0	0.0 ± 0.0	0.06
**Chest AIS**	3.0 ± 0.6	2.8 ± 0.8	2.7 ± 0.6	3.0 ± 0.0	0.67
**Abdomen AIS**	2.8 ± 1.0	2.9 ± 0.9	3.0 ± 0.8	3.6 ± 0.9	0.46
**ISS**	31.3 ± 15.4	24.5 ± 13.4	19.0 ± 8.9	21.6 ± 11.4	0.12
**Intubated ETT**	21(61.8%)	19 (67.9%)	1 (25.0%)	3 (60.0%)	0.42
**FAST** (Positive)	14 (41.2%)	13 (46.4%)	3 (75.0%)	4 (80.0%)	0.63
**Exploratory Laparotomy**	15 (44.1%)	15 (53.6%)	2 (50.0%)	5 (100%)	0.13
**Hospital length of stay**	11 (1–61)	16.5 (1–57)	36.5 (21–56)	19 (19–40)	0.03
**ICU length of stay**	5 (1–39)	10 (1–20)	12 (1–30)	7 (1–16)	0.88
**Ventilatory days**	6 (1–26)	8 (1–13)	30 (30–30)	3 (3–6)	0.26
**Blood Transfusion**	26 (76.5%)	20 (71.4%)	2 (50.0%)	3 (60.0%)	0.64
**Blood units transfused**	9.5 (2–26)	6 (1–32)	16.5 (1–32)	10 (10–16)	0.63
**Mortality**	14 (41.2%)	7 (25.0%)	1 (25.0%)	0 (0.0%)	0.21

### Glasgow Coma Scale on Admission

With regards to clinical parameters, 38% of patients had a GCS score of <9 (Table 3). Patients with a GCS <9 had a significantly higher ISS score (average 37 compared to 21.3 in patients with GCS >9, *p* = 0.001) and higher mortality.

**Table 3:** Pancreatic injury by Glasgow coma scale.

	**GCS ≥9 (*n* = 44)**	**GCS <9 (*n* = 27)**	***P*-Value**
**Hemorrhagic shock**	12 (27.3%)	13 (48.1%)	0.07
**Injury severity score**	21.3 ± 12.1	37.0 ± 12.7	0.001
**Pancreatic injury grade I-II**	36 (81.8%)	26 (96.3%)	0.07 for all
**Pancreatic injury grade III-IV**	8 (18.2%)	1 (3.7%)	
**Exploratory Laparotomy**	25 (56.8%)	12 (44.4%)	0.31
**Hospital length of stay**	17 (1–61)	9 (1–57)	0.04
**ICU length of stay**	5.5 (1–39)	9 (1–35)	0.81
**Ventilatory days**	8 (1–30)	7 (1–25)	0.58
**In-hospital complications**			
Wound Infection	9 (20.5%)	2 (7.4%)	0.14
Pneumonia	6 (13.6%)	5 (18.5%)	0.58
Acute respiratory distress syndrome	2 (4.5%)	3 (11.1%)	0.29
Sepsis	4 (9.1%)	5 (18.5%)	0.24
Acute renal failure	1 (2.3%)	2 (7.4%)	0.29
Multiorgan failure	1 (2.3%)	1 (3.7%)	0.72
Coagulopathy	1 (2.3%)	0 (0.0%)	0.43
**Mortality**	4 (9.1%)	18 (66.7%)	0.001

### On-Admission Patients' Hemodynamic Status

Table 4 shows pancreatic injury in patients with and without hemorrhagic shock. There were 27 patients with SBP <90 at presentation (38%). Patients with hemorrhagic shock had a significantly higher ISS and higher mortality. Pancreatic injury scales did not differ significantly in patients with and without shock.

**Table 4:** Pancreatic injury with and without shock.

	**No hemorrhagic shock (*n* = 46)**	**Hemorrhagic Shock (*n* = 25)**	***P*-Value**
**GCS** **≥9**	32 (69.9%)	12 (48.0%)	0.07 for all
**GCS** ** <9**	14 (30.4%)	13 (52.0%)	
**Injury severity score**	23.2 ± 13.6	34.6 ± 13.2	0.001
**Pancreatic injury grade I-II**	40 (87.0%)	22 (88.0%)	0.90 for all
**Pancreatic injury grade III-IV**	6 (13.0%)	3 (12.0%)	
**Exploratory laparotomy**	24 (52.2%)	13 (52.0%)	0.98
**Hospital length of stay**	16 (1–61)	11 (1–57)	0.44
**ICU length of stay**	5.5 (1–39)	8.5 (1–29)	0.55
**Ventilatory days**	8 (1–30)	6 (1–22)	0.43
**In-hospital complications**			
Wound Infection	6 (13.0%)	5 (20.0%)	0.43
Pneumonia	4 (8.7%)	7 (28.0%)	0.07
Acute respiratory distress syndrome	5 (10.9%)	0 (0.0%)	0.08
Sepsis	5 (10.9%)	4 (16.0%)	0.53
Acute renal failure	3 (6.5%)	0 (0.0%)	0.19
Multiorgan failure	2 (4.3%)	0 (0.0%)	0.29
Coagulopathy	0 (0.0%)	1 (4.0%)	0.17
**Mortality**	10 (21.7%)	12 (48.0%)	0.02

### Injury Severity Score

Table 5 shows the pancreatic injury based on the ISS. Around 73% of patients had an ISS of equal or more than 16. All the mortality cases had ISS ≥16.

**Table 5:** Pancreatic injury by severity of injury.

	**ISS <16 (*n* = 19)**	**ISS ≥16 (*n* = 52)**	***P*-Value**
**GCS** **≥9**	2 (10.5%)	23 (44.2%)	0.004 for all
**GCS** ** <9**	17 (89.5%)	27 (51.9%)	
**Pancreatic injury grade I-II**	15 (78.9%)	47 (90.4%)	0.20 for all
**Pancreatic injury grade III-IV**	4(21.1%)	5(9.6%)	
**Hemorrhagic shock**	2 (10.5%)	23 (44.2%)	0.01
**Exploratory Laparotomy**	10 (52.6%)	27 (51.9%)	0.95
**Hospital length of stay**	19 (1–56)	13 (1–61)	0.45
**ICU length of stay**	4 (1–16)	8.5 (1–39)	0.09
**Ventilatory days**	3.5 (1–8)	8 (1–30)	0.17
**In-hospital complications**			
Wound Infection	4 (21.1%)	7 (13.5%)	0.43
Pneumonia	0 (0.0%)	11 (21.2%)	0.07
Acute respiratory distress syndrome	0 (0.0%)	5 (9.6%)	0.16
Sepsis	1 (5.3%)	8 (15.4%)	0.25
Acute renal failure	0 (0.0%)	3 (5.8%)	0.28
Multiorgan failure	0 (0.0%)	2 (3.8%)	0.38
Coagulopathy	0 (0.0%)	1 (1.9%)	0.54
**Mortality**	0 (0.0%)	22 (42.3%)	0.001

### Management

**Figure 3:**
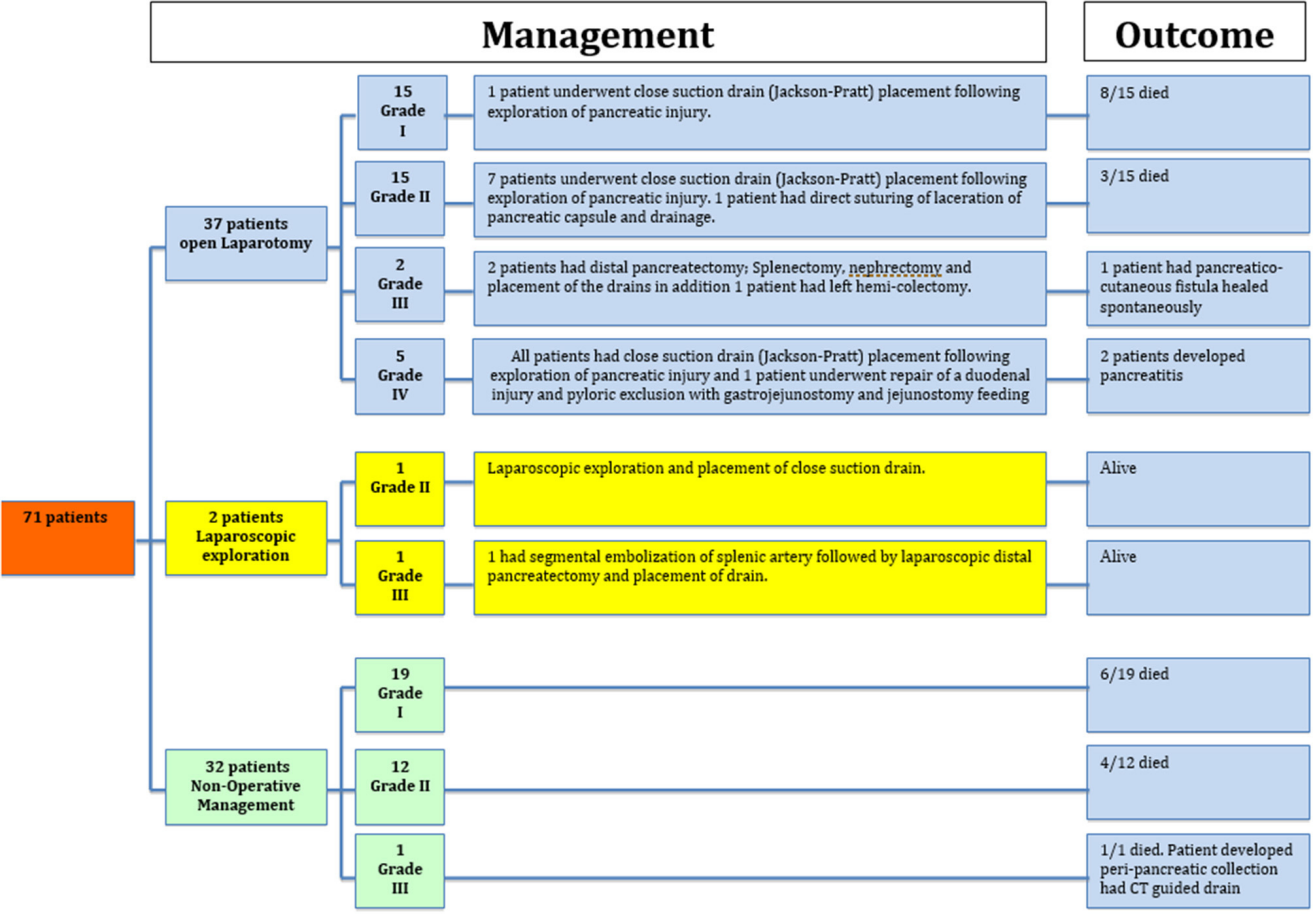
Management and outcomes based on the pancreatic injury grade.

Management and outcomes based on the injury grade are given in Figure 3.

A total of 37 patients underwent exploratory laparotomy (seven patients for pancreatic injuries [two grade III had a distal pancreatectomy and five grade IV had repair and drainage] and 30 patients had surgery for associated intra-abdominal injuries). In addition, two patients had laparoscopic exploration and drainage; one of them had distal pancreatectomy. NOM was used in 32 patients (19 had grade I and 12 had grade II and 1 had grade III).

### Mortality

Almost one-third of patients succumbed to their injuries (22/71). Mortality was not related to the grade of pancreatic injuries, but to the level of GCS, ISS score, and initial SBP. Most of the deaths in patients with low-grade pancreatic injuries were due to the associated brain injury and polytrauma. One patient had pancreatic-specific mortality in grade III (multiorgan failure), and no mortality was reported in patients with grade IV pancreatic injuries.

## Discussion

Data on pancreatic trauma are lacking in our region in the Arab Middle East. In this study, the prevalence of pancreatic trauma between total trauma admission (0.6%) and abdominal trauma (3.4%) is low and consistent with the concurrent literature (4, 12, 15). Blunt trauma is the most common type of pancreatic injury and over half of them are following road traffic accidents and about a quarter is related to fall from height. These results are in stark contrast to data from South Africa where over 70% of patients sustained penetrating injuries; most of them were due to gunshots (16).

The mortality rate, in the current study, was high but did not reflect the severity of the pancreatic injury and was mainly related to polytrauma, associated head injury, and the initial unstable hemodynamics. Hwang et al. analyzed 75 patients and showed that a GCS <13 was a significant predictor of mortality; and 47.6% of patients with GCS <13 had died (17).

The presence of hemorrhagic shock upon admission is an important predictor of mortality in pancreatic injury. Krige et al. and Hwang et al. had similar results, with a mortality rate of 29% and 35.1, respectively, in patients with systolic blood pressure <90 mmHg (16, 17).

In this study, low-grade (AAST I and II) pancreatic injuries accounted for nearly 90% of all injuries, similarly, Siboniet al. reported 83% of patients with low-grade injuries (18).

Shibahashi et al. and Gupta et al. showed nearly 40–50% of their patients had low-grade injuries (4, 12). However, Gupta et al. had a higher number of grade III injuries. Also, in a study by Krige et al. almost equal numbers of low- and high-grade injuries were reported (16). Although multiple studies have shown an association between pancreatic injuries grade and mortality rate, however, our study did not demonstrate a significant association (4, 16, 19).

The location of injury was evenly distributed throughout the pancreas in the present study.

Pancreatic injuries are rarely isolated and are commonly associated with other visceral or extra-abdominal injuries (20). In our study, the isolated pancreatic injury was found in eight patients (11.3%), while Siboni et al. and Shibahashi et al. reported 20 and 29.6% isolated injuries, respectively (4, 18). Traumatic head injury was the most common associated extra-abdominal injury in our cohort occurring in almost 43% of patients, followed by thoracic injuries in 27%. Pancreatic injury is commonly associated with another solid organ injury with the spleen being the most common injured organ (4, 7, 12, 16, 21, 22).

Serum amylase is normal at admission in up to 40% of patients with pancreatic trauma and is neither sensitive nor specific for definitive screening or diagnosis of pancreatic injuries, particularly within the first 3–6 h after injury. Serum lipase is more specific than amylase and more helpful for screening (23). Decreasing enzymes levels were found to be correlated with the success of NOM (21, 24, 25).

In this study, we observed a positive relationship between the grade of pancreatic injury and of the levels of pancreatic enzymes. However, this finding needs further support and explanation in prospective and larger studies. Moreover, the pancreatic enzymes could be elevated in patients with intra-abdominal or craniofacial injuries (23).

Mahajan et al. in a prospective study of 164 patients concluded that both pancreatic enzymes showed 85% sensitivity and 100% specificity, although this was time-dependent and was found to be significant when measured after 6 h of injury (21).

Focused assessment with sonography in trauma scan has a limited role in detecting solid organ injuries and with respect to the pancreas; the standard protocol of the examination does not cover its anatomical region (9, 26, 27).

Abdominal CT scans are the diagnostic modality of choice for pancreatic injury in hemodynamically stable patients, with a wide range of sensitivity. However, for detecting pancreatic duct injuries using a multidetector CT scan, the sensitivity had reached 91–95% with specificity up to 100% (11). Generally, pancreas may appear normal in 20–40% of the acute blunt injuries if imaging is done in the early 12-h post-trauma (25).

Operative management of pancreatic injuries depends on the grade of injury and associated injuries and can range from simple drainage for minor injuries to distal pancreatectomy and more complex reconstructive procedures and pancreaticoduodenectomy for extensive injuries (11).

Many studies showed that NOM for pancreatic trauma may be safe and effective in selected patients. The selection of patients for NOM is the key. It is widely acceptable that if the patient is stable with a low-grade injury, in the absence of an associated injury mandating explorative laparotomy, NOM should be attempted first (12, 28–31).

The recent World Society of Emergency Surgery guidelines recommended NOM for hemodynamically stable grade I and selected grade II pancreatic injuries (9). In high-level trauma center, NOM may be considered in selected hemodynamically stable patients with grade III pancreatic injuries that have proximal pancreatic body injuries without other abdominal injuries requiring surgery. NOM of grade IV injury is controversial and should only be attempted in highly specialized centers with adequate availability of high-quality intensive care facilities, endoscopy, and interventional radiology team (9, 28, 32). Shibahashi et al. had similar findings in their analysis where just over half of patients underwent laparotomy with higher grade injury associated with an increased need for operative intervention, however, they showed no superiority of the operative management over NOM (4). In a study by Krige et al. all patients underwent laparotomy surgery and most of them required only pancreatic drainage (mostly grade I and II pancreatic injuries), 111 patients had a distal pancreatectomy and 19 patients had pancreaticoduodenectomy (16).

Although NOM is a feasible option in most cases of low-grade pancreatic injuries, the failure of this approach will require subsequent surgery or delayed surgical intervention due to initially missed main pancreatic duct injury leading to higher pancreas-specific mortality (33).

In the present study, only eight patients (11%) had complications directly related to pancreatic injury and 75% of these patients had high-grade pancreatic injuries. Complications were more often in the higher grade injury groups. Intra-abdominal collections were the most common complication, followed by pancreatitis which developed in two patients; one of them developed a pancreatico-cutaneous fistula. Both patients with pancreatitis had grade IV injuries. One patient with grade III injury developed a pancreatic cyst. In the study by Al-Ahmadi et al., 38% of all patients developed non-endocrine complications and were more common in patients with blunt injury as opposed to penetrating trauma (22). Gupta et al. reported a complication rate of around 43% and most patients developed collections and pancreatitis (12). Krige et al. reported pancreas-related complication rate of almost 21% with a similar picture of more complications with high grades of injury (16). They also noted that age, presence of shock, the need for blood transfusion and the volume of blood transfused, damage control surgery, and the need for relook laparotomy and associated vascular injury were significant predictors of in-hospital complications.

### Limitations

This is a retrospective study, which is one of the limitations. Although the sample size is relatively small, it is representative of the country population as the data were abstracted from the nationwide trauma database in Qatar. This trauma registry has regular internal and external validation as it is linked to the National Trauma Database Bank (NTBD) in the USA. Our trauma center is the only level 1 tertiary trauma center in the country; it manages the moderate-to-severe trauma cases free of charge for all the country residents. This trauma center serves a population of 2.6 million (Qatar population) and receives an average of 1,600–1,800 trauma admissions annually. This study explores for the first time in our country the local experience in the diagnosis and management of traumatic pancreatic injury, which will help to improve our learning curve for early diagnosis and treatment of pancreatic injury. Of note, the long-term outcomes are lacking in this study. Also 69 out of the 71 patients with pancreatic injury were males and young (mean age 31 years). The country population is predominantly male, currently splits at 75% male and 25% female due to immigrant workers which constitute 85% of the total population^1^. Our previous works showed that 90% of the trauma patients were young males (34). Furthermore, another study from our institution showed that T1 trauma activation patients were more likely to present with higher ISS and 94% of them were males (35).

## Conclusion

Pancreatic injuries following abdominal trauma are uncommon, and the injured subjects are usually young male. However, most injuries are of low-grade severity. Radiologic and laboratory findings of acute pancreatic injury may be subtle; however, the deep location in the retroperitoneal region makes its injury uncommon and its diagnosis more difficult. Accurate identification of pancreatic trauma, grading, associated injury, and patient stability is mandatory to set an appropriate treatment strategy. This study shows that shock, higher ISS, and lower GCS are associated with worse in-hospital outcomes regardless of the severity of the pancreatic injury. NOM may suffice in patients with lower grade injuries, which may not be the case in patients with higher grade injuries unless carefully selected. Larger prospective studies are warranted for better risk assessment and management.

## Data Availability Statement

The original contributions presented in the study are included in the article/supplementary material, further inquiries can be directed to the corresponding author/s.

## Ethics Statement

This retrospective study granted ethical approval from the medical research center and institutional review board of Hamad Medical Corporation, Doha, Qatar (IRB#14409/14& IRB # MRC-01-18-003) with a waiver of consent as there was no direct contact with patients and de-identified data were collected retrospectively.

## Author Contributions

HA-T, AR, AA-H, GS, and AE-M: conceptualization and writing—original draft preparation. HA-T, AA-H, and AE-M: methodology. AE-M: formal analysis and writing—review and editing. HA-T and AR: investigation. HA-T, AA-H, and AR: data curation. All authors have read and agreed to the published version of the manuscript.

## Conflict of Interest

The authors declare that the research was conducted in the absence of any commercial or financial relationships that could be construed as a potential conflict of interest.

## Publisher's Note

All claims expressed in this article are solely those of the authors and do not necessarily represent those of their affiliated organizations, or those of the publisher, the editors and the reviewers. Any product that may be evaluated in this article, or claim that may be made by its manufacturer, is not guaranteed or endorsed by the publisher.

## Footnotes

^1^https://worldpopulationreview.com/countries/qatar-population (accessed November 24, 2021).

## Abbreviations

NOM: non operative management
ISS: injury severity score
GCS: Glasgow coma scale
CT scan: computerized tomographic scanning
MRI: magnetic resonance imaging.
